# Feasibility of magnetomyography with optically pumped magnetometers in a mobile magnetic shield

**DOI:** 10.1038/s41598-024-69829-y

**Published:** 2024-08-16

**Authors:** Simon Nordenström, Victor Lebedev, Stefan Hartwig, Marlen Kruse, Justus Marquetand, Philip Broser, Thomas Middelmann

**Affiliations:** 1 Physikalisch-Technische Bundesanstalt, 10587 Berlin, Germany; 2 Hertie Institute for Clinical Brain Research, University of Tübingen, 72076 Tübingen, Germany; 3 Ostschweizer Kinderspital, 9006 Sankt Gallen, Switzerland

**Keywords:** Magnetic shields, Non-invasive muscle measurements, FEM simulations, QuSpin QZFM Gen. 3, Twinleaf MS-2, Biomagnetism, Applied physics, Neuromuscular disease, Biomedical engineering

## Abstract

While magnetomyography (MMG) using optically pumped magnetometers (OPMs) is a promising method for non-invasive investigation of the neuromuscular system, it has almost exclusively been performed in magnetically shielded rooms (MSRs) to date. MSRs provide extraordinary conditions for biomagnetic measurements but limit the widespread adoption of measurement methods due to high costs and extensive infrastructure. In this work, we address this issue by exploring the feasibility of mobile OPM-MMG in a setup of commercially available components. From field mapping and simulations, we find that the employed zero-field OPM can operate within a large region of the mobile shield, beyond which residual magnetic fields and perturbations become increasingly intolerable. Moreover, with digital filtering and moderate averaging a signal quality comparable to that in a heavily shielded MSR is attained. These findings facilitate practical and cost-effective implementations of OPM-MMG systems in clinical practice and research.

## Introduction

In clinical neurophysiology, measuring brain currents and the electrical activity of nerves and muscles is crucial for studying the respective functional processes in a time-resolved manner. Typically, for this purpose, the electrical potentials are measured on the surface of the skin (e.g., electroencephalography, EEG, or surface electromyography, s-EMG) or invasively in the tissue utilizing inserted needles (n-EMG) or implanted electrodes (electrocorticography). In the same way that the underlying electrical phenomena cause potential fluctuations on the skin’s surface, they also effect magnetic field variations. Although the latter are exceptionally small, being on the order of fT in the brain and pT in the muscles, they can be measured with eminently sensitive measurement methods. However, magnetic field noise of a few to potentially several hundred or more $\hbox {pT}/\sqrt{\hbox {Hz}}$ is typically present in unshielded environments at the frequency bands of interest in biomagnetism^1–4^. To measure biomagnetic activity or other weak magnetic signals in unshielded or weakly shielded environments, researchers must often resort to averaging over many repetitions, complex signal processing, differential or gradiometric sensor arrangements^4,5^, and active coil compensation systems^6–8^. Therefore, biomagnetic measurements are most often performed in magnetically shielded rooms (MSRs). Traditionally, superconducting quantum interference devices (SQUIDs) have been employed to record these minute magnetic fields. While low critical temperature (low-Tc) SQUIDs offer an unrivaled combination of sensitivity (sub-$\hbox {fT}/\sqrt{\hbox {Hz}}$) and bandwidth ($\hbox {MHz}$ range)^9^, they are large and inflexible due to the required cryogenic cooling. Furthermore, low-Tc SQUID arrays entail a large cold-warm distance, typically of a few centimeters. High-Tc SQUIDs enable a shorter cold-warm distance^10^, at the expense of higher noise levels, but remain bulky and inflexible.

More recently, new sensing technologies have been developed for biomagnetism, among which are magnetoresistive^11^ and magnetoelectric^12,13^ sensors, and optically pumped magnetometers (OPMs) based on atomic vapors^14–16^ or color centers in crystals^17^. While all of these can be manufactured as compact and small sensor heads, allowing flexible placement and combination in arrays of sensors, only OPMs already provide sufficient sensitivity to capture biomagnetic signals *in vivo* (${\ll }\hbox {1 pT}/\sqrt{\hbox {Hz}}$) in magnetically shielded environments without averaging. Furthermore, with such compact sensors, the required size of the magnetic shielding is now mainly determined by the specific application. Thus, in the case of magnetomyography (MMG) on the limbs, a mobile shield might be sufficient^18^.

The history of MMG is about as old as the one of magnetoencephalography (MEG)^19^ and started with the employment of SQUIDs^20^. Since then, MEG has developed into an important field of research, with over 100 SQUID-MEG systems installed worldwide today. Mainly due to the lack of flexibility in sensor placement, MMG has not evolved much for many years and gained renewed interest^21^ only recently when highly sensitive and compact zero field (ZF) OPMs became commercially available for the first time^22–28^. These proof-of-concept studies have demonstrated the promising potential of OPM-MMG in physiological analyses of muscles and nerves, including the analysis of nerve innervation patterns of the human limbs and their extremities^23,25^, and the detection of pathological signs such as muscle fasciculations^22^ in patients with known neuromuscular disorders. However, they were all performed in magnetically shielded rooms. For MMG to gain widespread adoption in clinical settings, designated mobile measurement setups must be developed and their performance analyzed, similar to emerging mobile MEG systems^29,30^. Iwata et al.^18^ have recently shown that OPM-MMG may indeed be performed in a clinical environment using only a table-top shield. Due to the sizeable opening in the shield required for arm access, however, some ZF-OPMs malfunctioned due to large environmental fields. Moreover, the signal quality was comparatively poor and it is not clear to what extent the muscular signal was distorted by surrounding noise and perturbations. The feasibility of mobile OPM-MMG thus remains a vastly unexplored topic.

In this study, we aim to qualitatively and quantitatively compare the typical conditions for MMG in a commercially available mobile magnetic shield with those in a heavily magnetically shielded room. First, we investigate the noise and perturbations present in the two shielded environments. Second, through simulations and field mapping, we determine the region within the mobile shield in which commercial ZF-OPMs can operate without the residual magnetic field being too high. Third, we compare the MMG signals obtained from the evoked response of the abductor digiti minimi (ADM) muscle of the hand to electrical ulnar nerve stimulation in the respective shielded environments.

## Methods

### Triaxial ZF-OPM

Optically pumped magnetometers utilize the interaction of atoms with magnetic fields and light to measure magnetic fields with high sensitivity. In particular, the commercially available triaxial ZF-OPM (QZFM Gen. 3, QuSpin Inc.) employed in this study exploits spin-polarized atoms and the so-called spin-exchange-relaxation-free regime (SERF) to achieve ultra-high magnetic field sensitivity. The sensor has a specified sensitivity of $< {23}\,\hbox {fT}/\sqrt{\hbox {Hz}}$ from ${3}\,{\hbox {Hz}}$ up to ${100}\,{\hbox {Hz}}$, and a 3-dB bandwidth of ${135}\,{\hbox {Hz}}$^31^. A permanently built-in 6th-order digital lowpass filter with a 500 Hz cut-off frequency suppresses the sensor response at frequencies substantially above the nominal bandwidth^32^. The minimal distance between the sensing element’s center and the housing is about ${5}\,{\hbox {mm}}$, allowing for low standoffs. A near zero-field environment is required for sensor operation and is typically realized with external magnetic shielding. In addition, sensor-internal compensation coils can null small residual fields up to, nominally, ${50}\,\hbox {nT}$. Two separate light beams together with magnetic field modulation at ${923}\,{\hbox {Hz}}$ along each axis allow for triaxial measurements by phase-sensitive lock-in demodulation^33^. As the output signal of the sensor is a dispersive function of the field, some non-linearity is present (practically, ${< 5}{\%}$ field deviation within a dynamic range of ${\pm 1}\,\hbox {nT}$).

### OPM-MMG of the ADM

**Figure 1:**
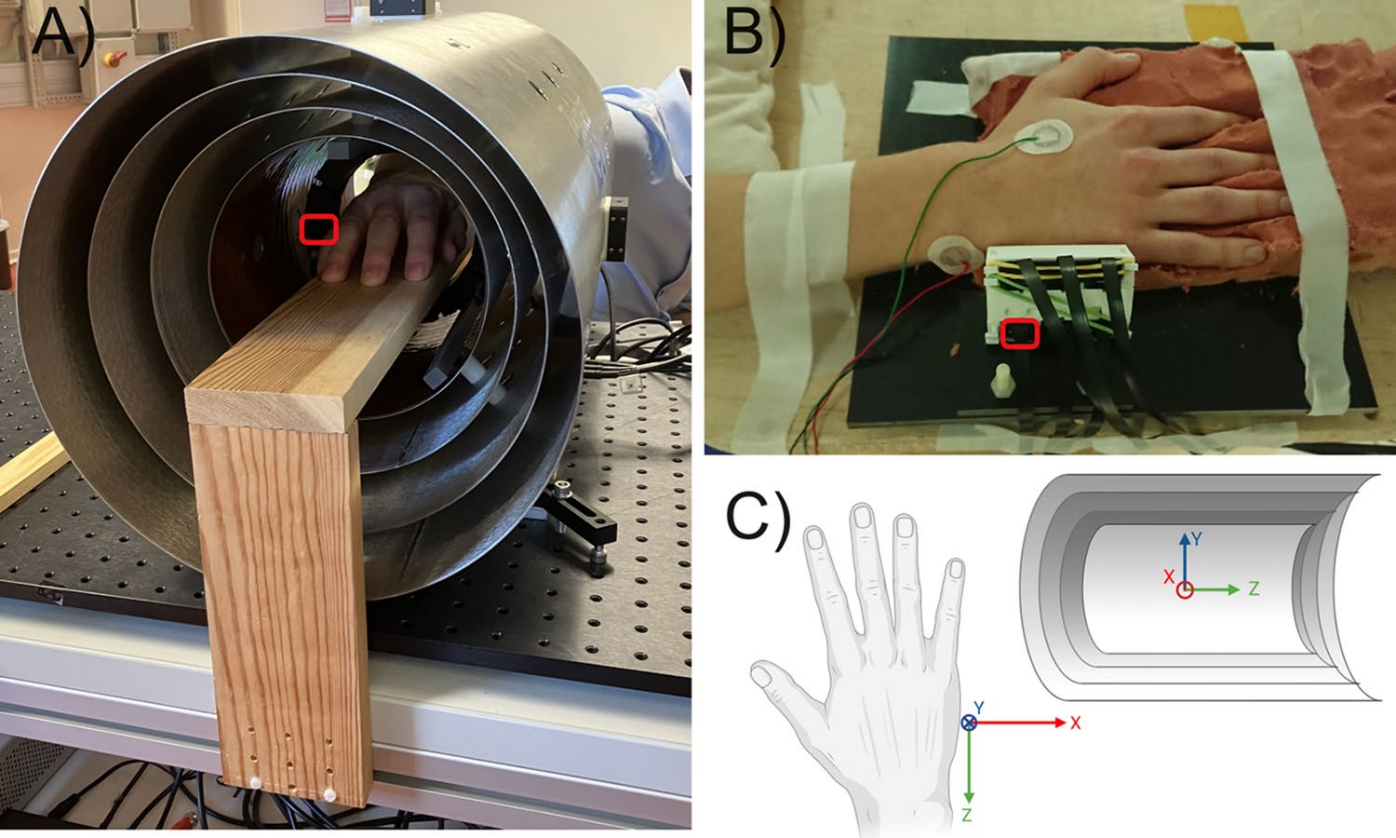
Experimental setup in mobile shield MS-2 (**A**) and magnetically shielded room BMSR-2 (**B**). Red rectangles mark the OPM position. The EMG electrodes visible in (**B**) were not used in this study. (**C**) The coordinate systems used in the MMG experiments and the field mapping and simulation. Created with BioRender.com.

The experimental setups are shown in Fig. 1. The abductor digiti minimi (ADM) muscle of the hand was chosen as the muscle of interest due to its comparatively isolated location, which minimizes signals from nearby muscles, the ease of electrically stimulating it at a distant position, and its clinical relevance^34–36^. The ulnar nerve was electrically stimulated (Micromed, Venice, Italy) at the cubital tunnel. Stimulation of the ulnar nerve evokes a reproducible muscle response of the ADM that may be measured magnetically using highly sensitive magnetometers. A monopolar electrical pulse with a duration of ${0.1}\,\hbox {ms}$ at a repetition rate of ${1}\,{\hbox {Hz}}$ was used for stimulation, and the triaxial OPM was positioned lateral to the ADM to measure the muscle activity. The stimulation current was set to produce a visually discernible muscle twitch. The distance from the center of the OPM’s vapor cell (i.e., the sensing element) to the skin was approximately ${10}\,\hbox {mm}$. A trigger signal from the stimulator device enabled time-locked averaging of the MMG signals. The participants are authors of this paper and gave informed consent before participating in the study. The study was conducted in accordance with the Declaration of Helsinki and approved by the ethics committee of the University of Tübingen.

### Setup and procedure in mobile shield

The commercially available magnetic shield (MS-2, Twinleaf LLC), consisting of four nested cylinders of mu-metal, was positioned on a mobile wagon (Fig. 1A). The endcaps on both sides were removed to allow for arm access. To orient the shield transversely to the geomagnetic field, the wagon was rotated while the magnetic field was simultaneously measured inside the shield with a fluxgate (Mag690-100, Bartington). The subject’s arm (male, 35 years old, healthy) was placed on a wooden plank within the shield. Mechanical decoupling was ensured by vibrational isolators (eight Sorbothane bumpers) between the breadboard on which the magnetic shield was resting and the rest of the wagon to which the wooden plank was attached. The OPM was positioned next to the ADM in a 3D-printed circular holder connected to the innermost cylinder of the shield and the MMG signal from electrical ulnar nerve stimulation was recorded for ${60}\,\hbox {s}$. Subsequently, the arm was withdrawn from the shield and a noise measurement was performed with endcaps off. The coordinate system of the hand in Fig. 1C corresponds to the OPM’s innate coordinate system, and the MS-2 coordinate system is identical up to the signs of the components and the position of the origin. Finally, the frequency response of the OPM was obtained by supplying up-chirp signals to the built-in MS-2 coils, with the endcaps on and the sensor positioned in the center of the shield. The analog output data from the OPM were sampled at 5 ksps with a 24-bit resolution data acquisition system (National Instruments, PXI-4462).

### Setup and procedure in magnetically shielded room

The Berlin magnetically shielded room 2 (BMSR-2) comprises eight mu-metal layers and one aluminum layer enclosed in a radiofrequency shield. The shielding layers additionally include coils for degaussing and active compensation of low-frequency magnetic field drifts. A room of volume ${2.9}\,\hbox {m}$ $\times$ ${2.9}\,\hbox {m}$ $\times$ ${2.8}\,\hbox {m}$ (width, depth, height) is accessible for experiments inside the innermost layer. The residual field in the center of this room is typically ${< 1}\,\hbox {nT}$ whereas the residual field gradient is $< {2}\,\hbox {nT}/\hbox {m}\;$^37^. The subject’s hand (male, 26 years old, healthy) was stabilized in orthopedic foam (not used in the mobile setup due to spatial constraints) on a table inside the BMSR-2 (Fig. 1B). Six OPMs were placed in a 3D-printed holder next to the ADM. For the purposes of this paper, we only analyze the same OPM that was used in the mobile setup experiment (a separate paper on grid analysis and EMG recordings will be published soon). A coordinate transformation was carried out to go from the innate OPM coordinate system to the one of the hand shown in Fig. 1C (${x\rightarrow z}$, ${ y \rightarrow y}$, ${ -z \rightarrow x}$). Due to the low magnetic field gradients in the room and the massive table, no additional measures were taken to optimize mechanical decoupling apart from the subject’s hand not touching the sensor array. The ulnar nerve was stimulated 7 times, and a noise measurement was performed with the stimulator turned off. The analog output data from the OPM were sampled at 4 ksps with the 24-bit data acquisition system of the BMSR-2 (LanDAQ, Lay-Audio Systeme).

### Data processing

Post-processing was executed in Wolfram Mathematica 13.2. The MMG data were bandpass filtered using a zero-phase, sixth-order Butterworth filter at cut-off frequencies of ${10}\,{\hbox {Hz}}$ and ${300}\,{\hbox {Hz}}$. For both data sets, zero-phase notch filtering was applied at ${50}\,{\hbox {Hz}}$ (filter width of ${2}\,{\hbox {Hz}}$). For the mobile setup data, additional notch filtering was performed at higher harmonics up to and including ${250}\,{\hbox {Hz}}$. Fifty-eight and seven epochs (single-shot signals) were used for averaging in the mobile setup and BMSR-2, respectively. MMG signal root-mean-square (RMS) was calculated within a ${14}\,{\hbox {ms}}$ segment starting at ${10}\,\hbox {ms}$ (including intrinsic sensor delay) after the stimulation trigger. Similarly, to obtain a metric for the quality of the unaveraged waveforms, the average root-mean-square deviation (RMSD) of single-shot signals from the averaged signal was computed within the same ${14}\,\hbox {ms}$ temporal window. Further, to explore the effects of digital filtering, the RMSDs were computed for unfiltered, bandpass filtered, and bandpass and notch filtered data separately. The RMSD values were put in relation to the signal of interest by additionally calculating normalized RMSD (nRMSD) values, i.e., the percent of deviation compared to the respective averaged signal peak-to-peak. Noise spectral densities (NSDs) were estimated from the noise measurements using Bartlett’s method (to decrease variance) with ${1}\,\hbox {s}$ data segments, and spectral leakage was reduced by applying Hann windows. The noise measurements were also used to estimate the RMS noise levels after averaging. The measured and applied chirp signals were Hilbert transformed and subsequently moving average smoothed to obtain the frequency response of the sensor.

### Field simulation and mapping of mobile shield

Finite element method (FEM) simulations of the static field distribution inside the table-top shield due to the surrounding geomagnetic field were carried out in the AC/DC module of COMSOL Multiphysics 5.6. A relative permeability of 28000 was used for the mu-metal. To approximately account for the laboratory conditions, the longitudinal axis of the shield was rotated by ${1}^\circ$ from the optimal direction (i.e., transverse to the incoming field) for comparison with the experimental data. Sextic polynomials were fitted to the three components of the FEM results with an adjusted ${R^2 > 0.99}$. The inclination (${69}^\circ$) and magnitude (${45}\,{\upmu }{\hbox {T}}$) of the geomagnetic field at the shield’s location were measured with the triaxial fluxgate magnetometer and incorporated in the simulations. The residual field was then experimentally mapped by manually translating the fluxgate within the shield.

## Results and discussion

### Characterization of sensor and environment

**Figure 2:**
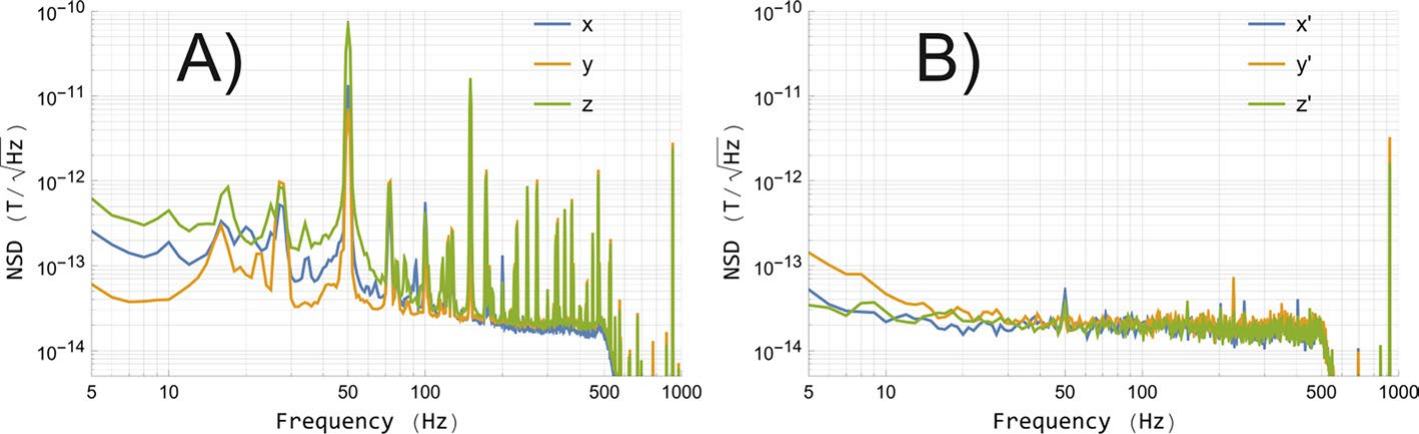
Noise spectral densities (NSDs) in the open MS-2 (**A**) and in BMSR-2 (**B**). The BMSR-2 components have been transformed from the sensor’s innate coordinate system to the one relative to the muscle in Fig. 1C.

**Figure 3:**
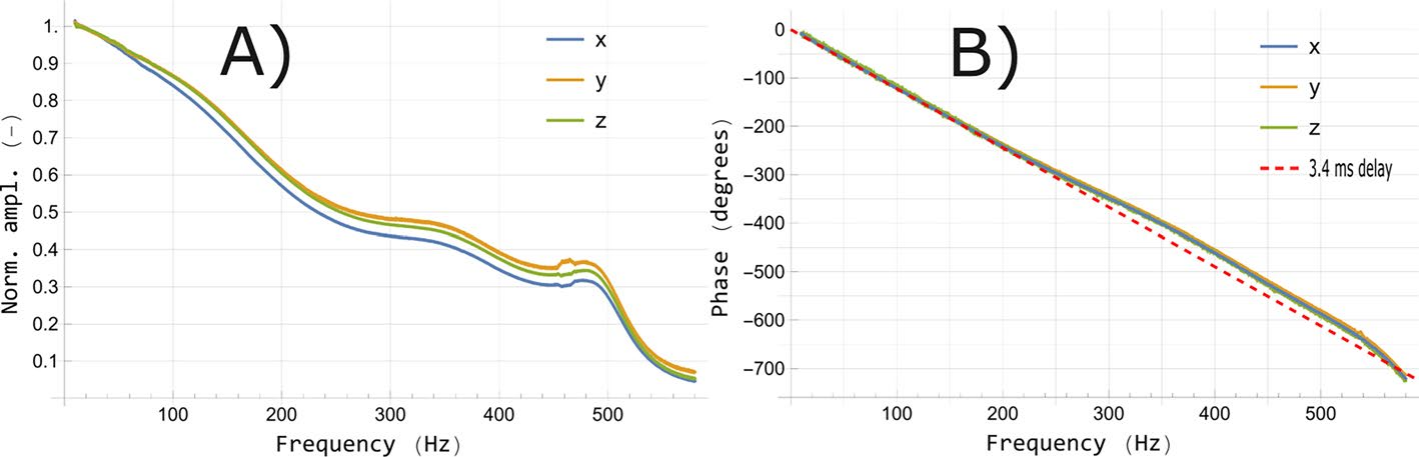
Frequency response of amplitude (**A**) and phase (**B**) of the employed OPM.

The results of the noise measurement from the mobile shield can be seen in Fig. 2A. Considering frequencies above ${10}\,{\hbox {Hz}}$ and disregarding distinct peaks from disturbances, the noise level here is approximately ${< 100}\hbox { fT}/\sqrt{\hbox {Hz}}$ in the X direction, ${< 70}\hbox { fT}/\sqrt{\hbox {Hz}}$ in the Y direction, and ${< 300}\hbox { fT}/\sqrt{\hbox {Hz}}$ in the Z direction. As the Z component is longitudinal to the MS-2 shield, one indeed expects the most magnetic noise along Z due to the large openings (however, this also depends on the position within the shield as the magnetic field lines bend). In contrast, the noise level in BMSR-2 in Fig. 2B is ${< 20}\hbox { fT}/\sqrt{\hbox {Hz}}$ above approximately ${20}\,{\hbox {Hz}}$ for all three components. Whereas the BMSR-2 data is virtually devoid of perturbations, numerous high-amplitude peaks are apparent in the MS-2 data. The sharp attenuation above ${500}\,{\hbox {Hz}}$ due to sensor-internal digital lowpass filtering and the magnetic field modulation at ${923}\,{\hbox {Hz}}$ are observed in both measurements.

In Fig. 3A, the amplitude frequency response of the sensor is presented. It qualitatively resembles that in^38^ where the same type of commercial OPM in the dual-axis variant was used. We attribute the slightly steeper slope at low frequencies in our measurement to typical variations between the sensors. A separate reference measurement with the fluxgate magnetometer showed no visible frequency dependence of the up-chirp amplitude. Depending on the axis, the 3-dB cut-off is between ${150}\,{\hbox {Hz}}$ and ${160}\,{\hbox {Hz}}$ (nominally ${135}\,{\hbox {Hz}}$). Based on typical bandwidths for various muscle signals and EMG modalities, the frequency band of interest in MMG ranges from approximately ${10}\,{\hbox {Hz}}$ up to potentially several kHz^39,40^. The low bandwidth of this and similar commercial ZF-OPMs is therefore a limiting factor for MMG. Importantly, the complex irregular shape of the amplitude response spectrum also distorts broadband signals, such as those originating from muscles, in a non-linear fashion. The bandwidth could be increased and the frequency response linearized by, for instance, using feedback techniques to lock to the center of resonance. However, this comes with a loss in sensitivity and a frequency-dependent increase in noise^16^. To a certain degree, the distortion may also be corrected by applying an appropriate (e.g., corresponding to the inverse of the amplitude response) filter to the output signal. Similarly to a non-linear amplitude response, a non-linear phase response would induce frequency-dependent time delays and thus signal distortion. Fortunately, the phase response in Fig. 3B of the OPM is comparatively linear and corresponds to a ${3.4}\,\hbox {ms}$ frequency-independent delay within the bandwidth. Since a lowpass filter with an upper cut-off frequency of ${300}\,{\hbox {Hz}}$ was used in the post-processing of the MMG signals, some non-linearity in the phase response is present.

### Simulation and mapping of residual field

**Figure 4:**
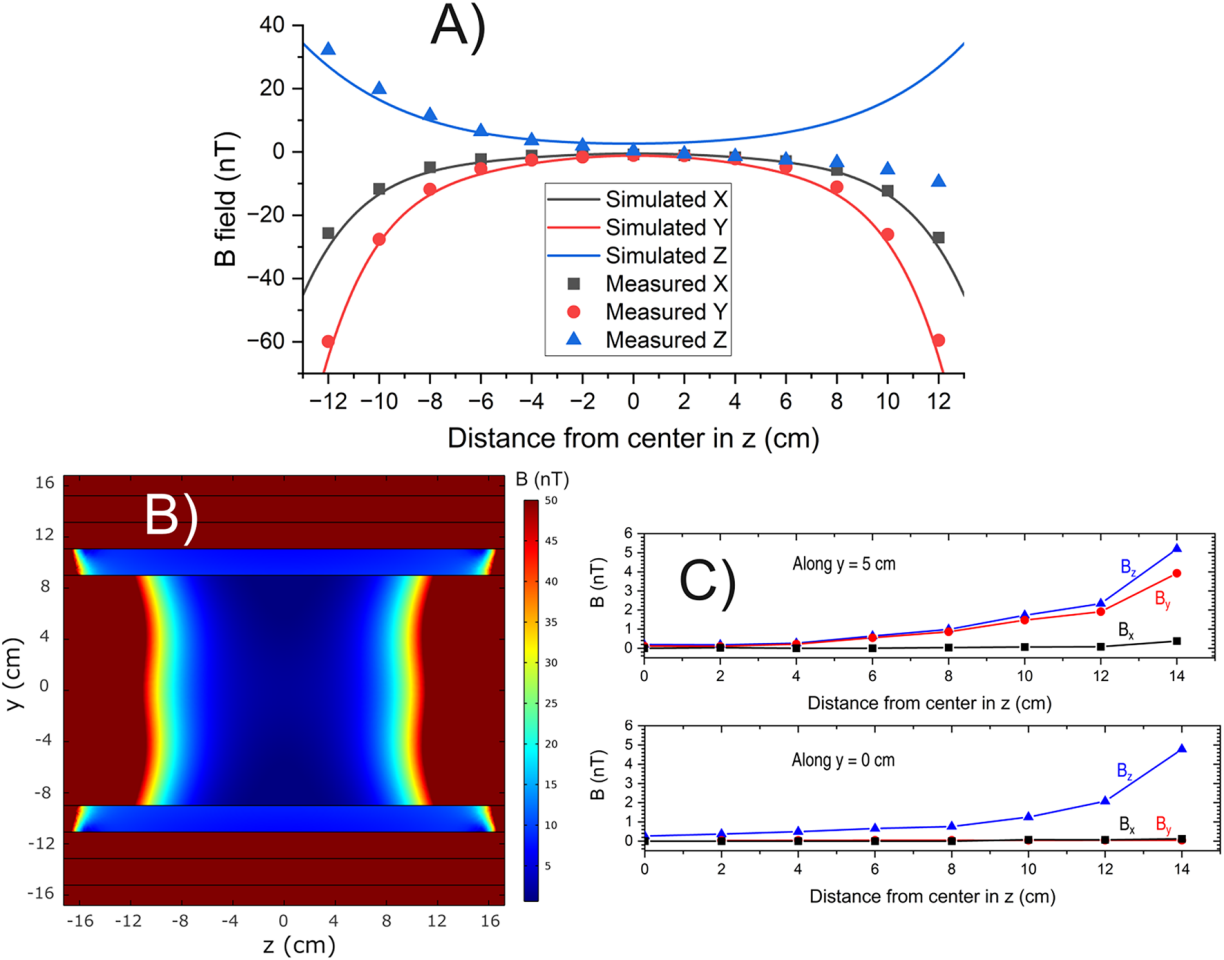
FEM simulations and experimental field mapping of the magnetic field within the MS-2 shield. (**A**) X, Y, and Z components of the static magnetic field. Note that the shield was rotated by one degree around the Y axis in the simulation to roughly emulate experimental conditions. Solid lines and discrete points represent simulations and experimental data, respectively. (**B**) Simulated magnitude of the static B field in the YZ plane. The data range is cut at ${50}\,\hbox {nT}$ to evince the zone where the ZF-OPM is expected to be operational. (**C**) Measured magnetic field of the power line at ${50}\,{\hbox {Hz}}$ along two longitudinal lines within the shield (here, solid lines only as a visual aid).

The simulated and experimental X (horizontal), Y (vertical), and Z (longitudinal) components of the residual DC field along the central line of the MS-2 are shown in Fig. 4A. The discrepancy between simulation and experiment is marginal in the X and Y components. However, the measured Z component is noticeably asymmetric and deviates more considerably from the simulation. It should be noted that a longitudinal magnetic DC component along the center line of the shield can only be present if the geomagnetic field lines are not completely transverse to the shield (in the simulation, the 1-degree rotation gives rise to the Z component). The additional asymmetry suggests non-uniformity of the magnetic field in our laboratory (i.e., “non-parallel” field lines) likely due to nearby magnetic objects. It is important to note that the DC axial shielding factor of the MS-2 with endcaps removed is only on the order of 100 according to simulations (assuming a perfectly degaussed shield, i.e., “quasi-DC”), which is more than three orders of magnitude lower than the shielding factor using endcaps (Supplementary Fig. S1). Thus, careful rotation of the shield in the horizontal plane is crucial for zero-field OPMs to work properly within a large region of the shield. From the simulated magnitude plot of an optimally oriented open MS-2 shield, Fig. 4B, we can conclude that the residual field is less than ${50}\,\hbox {nT}$ over a region of approximately ${\pm 10}\,\hbox {cm}$ from the shield’s center plane $(z = 0)$. Beyond this region, the internal coils of the OPM employed here are not expected to null the residual field successfully. The amplitude of the ${50}\,{\hbox {Hz}}$ component of the penetrating field in MS-2, as measured with the fluxgate, can be seen in Fig 4C. To ensure less than ${5}{\%}$ non-linearity due to large dynamic fields, of which in our case field perturbation due to the room’s power line at ${50}\,{\hbox {Hz}}$ is dominating, the OPM should not be placed beyond roughly ${12}\,\hbox {cm}$ from the center of the shield. This, however, depends on several factors such as the sources of the perturbations, the sensor’s position within the shield, and the component one wishes to analyze (compare the center line to the ${5}\,\hbox {cm}$ elevated line in Fig 4C). In addition to non-linearity, residual AC (DC) fields may also cause time-dependent (static) “cross-axis leakage”, effectively rotations of the sensitive axes, which to a certain degree falsifies the sensor readings^41,42^. Further research is called for to quantify the implications of such cross-axis leakage for MMG where commercial ZF-OPMs are utilized. In future studies, incorporating active compensation with coils through feedback from OPMs or other magnetometers could attenuate low-frequency perturbations and greatly cancel remnant fields in the MS-2. This could improve the low-frequency performance of the mobile setup and mitigate unwanted effects such as non-linearity and cross-axis leakage described above but at the cost of greater system complexity^43^.

### MMG signals

**Figure 5:**
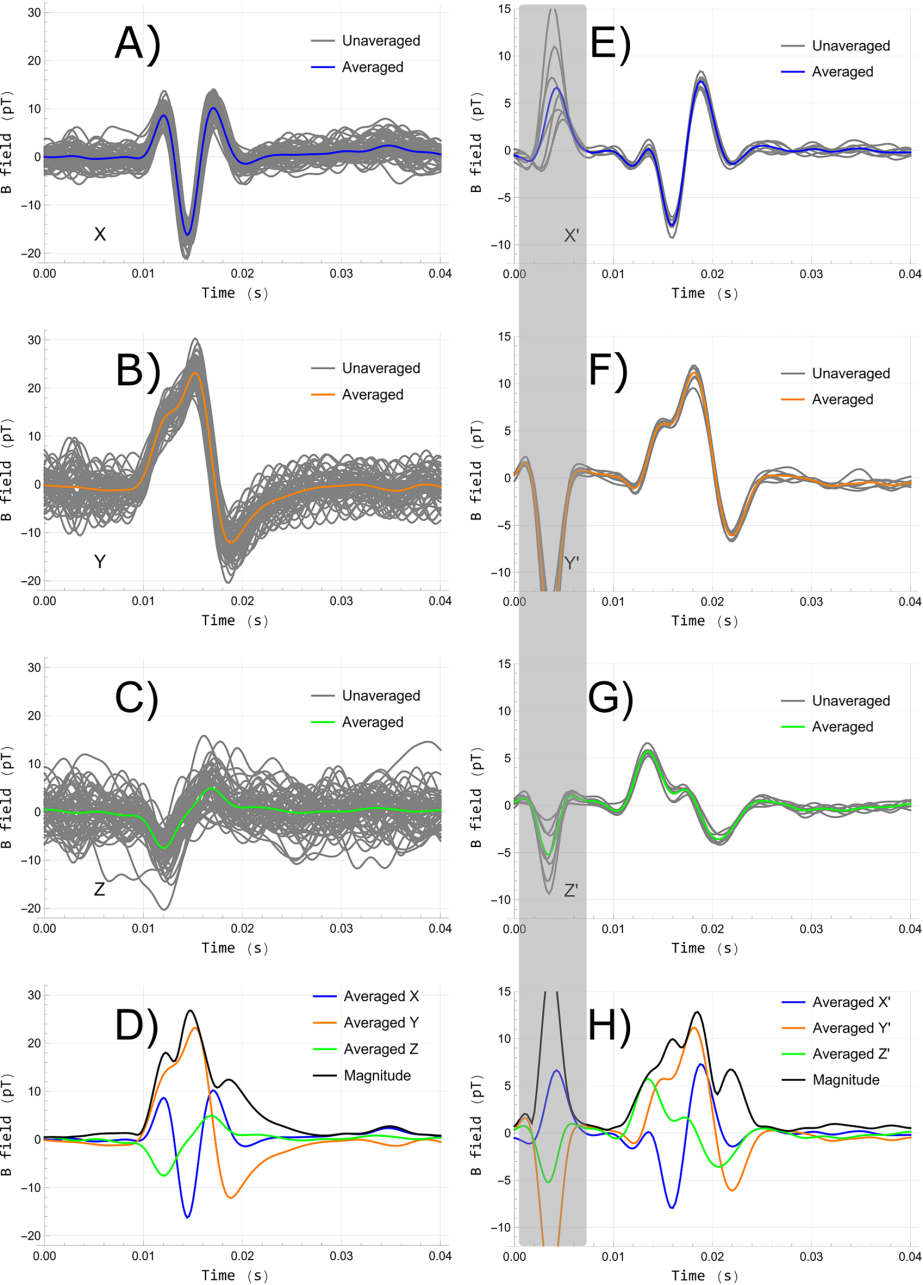
Unaveraged and averaged MMG signal components and magnitude in MS-2 (**A**–**D**) and BMSR-2 (**E**–**H**). Please note the gray vertical band highlighting the stimulation artifact present in the BMSR-2 recordings (shifted and blurred due to the sensor’s frequency response and additional digital filters). The MMG signals begin at approximately 10 ms including sensor delay.

In Fig. 5 the recorded MMG signals in MS-2 (the mobile setup) and BMSR-2 (the magnetically shielded room) are shown to the left and right, respectively. A quantitative comparison can be found in Table 1. Several differences and similarities are evident. First, the single-shot MMG signals in BMSR-2 are nearly identical to their average in all three projections. The RMSDs normalized to the peak-to-peak of the averaged signals (nRMSDs) are merely ${3}{\%}$ to ${5}{\%}$, approximately. In contrast, in MS-2 the unaveraged signals oscillate around their average due to a higher level of noise and perturbations (nRMSDs of ${8}{\%}$ to ${25}{\%}$). Nevertheless, the averaged signals show qualitatively similar waveforms in both experimental environments and the estimated RMS noise level is below ${1}\,\hbox {pT}$ for all components within the ${10}\,{\hbox {Hz}}$ to ${300}\,{\hbox {Hz}}$ passband after averaging. Moreover, single-shot signals may be sufficient even in this portable environment to estimate certain parameters that have shown diagnostic value in EMG, e.g., the number of phases (defined as the number of baseline crossings plus one) and signal amplitude^44^.

**Table 1:** Root-mean-square values of MMG signals, deviations (RMSD) of single-shot signals from averaged signals, and noise levels. Normalized (to signal peak-to-peak) RMSD is given in parentheses. UF: unfiltered. BP: bandpass filtered (${10}\,{\hbox {Hz}}$ to ${300}\,{\hbox {Hz}}$). NF: notch filtered (${50}\,{\hbox {Hz}}$. In MS-2 also higher harmonics). AV: averaged (MS-2: 58 averages. BMSR-2: 7 averages).

MMG component	Signal RMS (pT)		RMSD (pT)						RMS noise level (pT)	
			***UF***		***BP***		***BP+NF***		***BP+NF+AV***	
	MS-2	BMSR-2	MS-2	BMSR-2	MS-2	BMSR-2	MS-2	BMSR-2	MS-2	BMSR-2
X (radial)	6.5	3.7	11.4 (43.3 %)	2.0 (12.9 %)	11.1 (42.2 %)	0.48 (3.2 %)	2.15 (8.2 %)	0.48 (3.2 %)	0.41	0.12
Y (tangential)	12.2	5.6	8.7 (24.5 %)	3.3 (19.0 %)	8.2 (23.2 %)	0.50 (2.9 %)	2.91 (8.2 %)	0.50 (2.9 %)	0.73	0.14
Z (longitudinal)	3.4	2.7	61.0 (489 %)	1.4 (14.8 %)	60.9 (489 %)	0.43 (4.6 %)	3.08 (24.7 %)	0.42 (4.6 %)	0.71	0.12

Second, the onset (i.e., the time between stimulation and initiation of the muscle action potential) of the averaged MMG signals is qualitatively similar in both experiments. This reinforces Iwata et al.’s^18^ finding that the latency can be reliably estimated in a compact OPM-MMG setup without substantial averaging. From the latency and the distance from the stimulation point to the muscle, the nerve conduction velocity (NCV) can be estimated by knowledge of the effective synaptic transmission time at the neuromuscular junctions (${\approx 1}\,\hbox {ms}$) and the intrinsic sensor delay (${3.4}\,\hbox {ms}$, see Fig. 3B). In both the mobile setup and in BMSR-2, the estimated NCV of the ulnar nerve of the subjects is then on the order of ${55}\,\hbox {m}/\hbox {s}$ to ${65}\,\hbox {m}/\hbox {s}$, which is within the physiologically normal range^45^. A more accurate assessment of the NCV can be done by, for instance, additionally stimulating distally at the wrist. The NCV is crucial in electroneurography for evaluating patients with possible or known nerve damage.

Third, the stimulation artifact is visible in BMSR-2 but only faintly in MS-2. This possibly suggests that the magnetic stimulation artifact mainly originates from the electromagnetic field radiated from the stimulation electrodes and wires, which gets attenuated by the mu-metal before reaching the sensor in MS-2 as the stimulation electrodes are attached to the elbow outside the MS-2 shield. However, further investigation of the nature of the stimulation artifact in MMG is warranted.

Importantly, and as seen in Table 1, notch-filtering is pivotal in the mobile setup, whereas bandpass filtering is more effective in BMSR-2 due to low-frequency drifts and white noise dominating the noise (compare Fig. 2). Ideally, no notch filtering should be applied, as then also portions of the signal of interest will be filtered out^46^. Several alternative signal processing techniques exist that may allow for less distortion of the signal of interest^47,48^. Moreover, our simulations suggest that by adding endcaps to MS-2 with an access hole size adapted to the arm, approximately two orders of magnitude in both axial and transverse quasi-DC shielding factors can be expected (Supplementary Fig. S1), which likely enables highly reproducible single-shot measurements.

A limitation of this study of the MMG signals is the lack of multi-subject investigation, and the subject analyzed in MS-2 was not the same as in BMSR-2. Factors such as inter-subject anatomical variability^27^, degree of electrode-skin contact, health status, body temperature, and the exact sensor positioning and standoff to the effective field sources influence the amplitude, timing, and structure of the MMG signals, likely on the order of the differences observed between the measurements in MS-2 and BMSR-2 in Fig. 5. Nevertheless, this paper provides an analysis of typical MMG signals and environmental conditions one expects to encounter in similar shielded environments.

## Conclusions

We have presented the first qualitative and quantitative comparison between OPM-MMG performed in a mobile magnetic shield and a magnetically shielded room. The large access holes in the mobile shield resulted in higher levels of noise and perturbations and thus more postprocessing, i.e., digital filtering and averaging, was required to achieve a signal quality comparable to that obtained in the magnetically shielded room. In addition, the magnetic field distribution within the mobile shield was experimentally mapped and numerically simulated to estimate the region in which the employed sensors can operate successfully. The FEM-based simulation was consistent with the experimental maps and supported the finding of the importance of precise positioning of the shield with respect to the environmental field.

The presented results enable the design of mobile MMG-dedicated setups and sensor configurations within well-defined operating regions by employing only off-the-shelf magnetic shielding and sensors. This is an important basis for planning and performing MMG measurements without the necessity of a magnetically shielded room, thus making the exploration of MMG with a comparatively small budget and in the absence of a large infrastructure more accessible. Future work will employ a custom-designed magnetic shield whose geometry makes it especially suited for MMG and which provides sufficient space for semi-compact lab-based OPM sensor heads.

### Supplementary Information

Supplementary Figure S1.

## Data Availability

The datasets used in this study are available from the corresponding author on request.
