# Supplementary figures and images for: Feasibility of magnetomyography with optically pumped magnetometers in a mobile magnetic shield
Source: Sci Rep. 2024 Aug 16;14:18960. doi: 10.1038/s41598-024-69829-y (PMC11327291; doi:10.1038/s41598-024-69829-y)

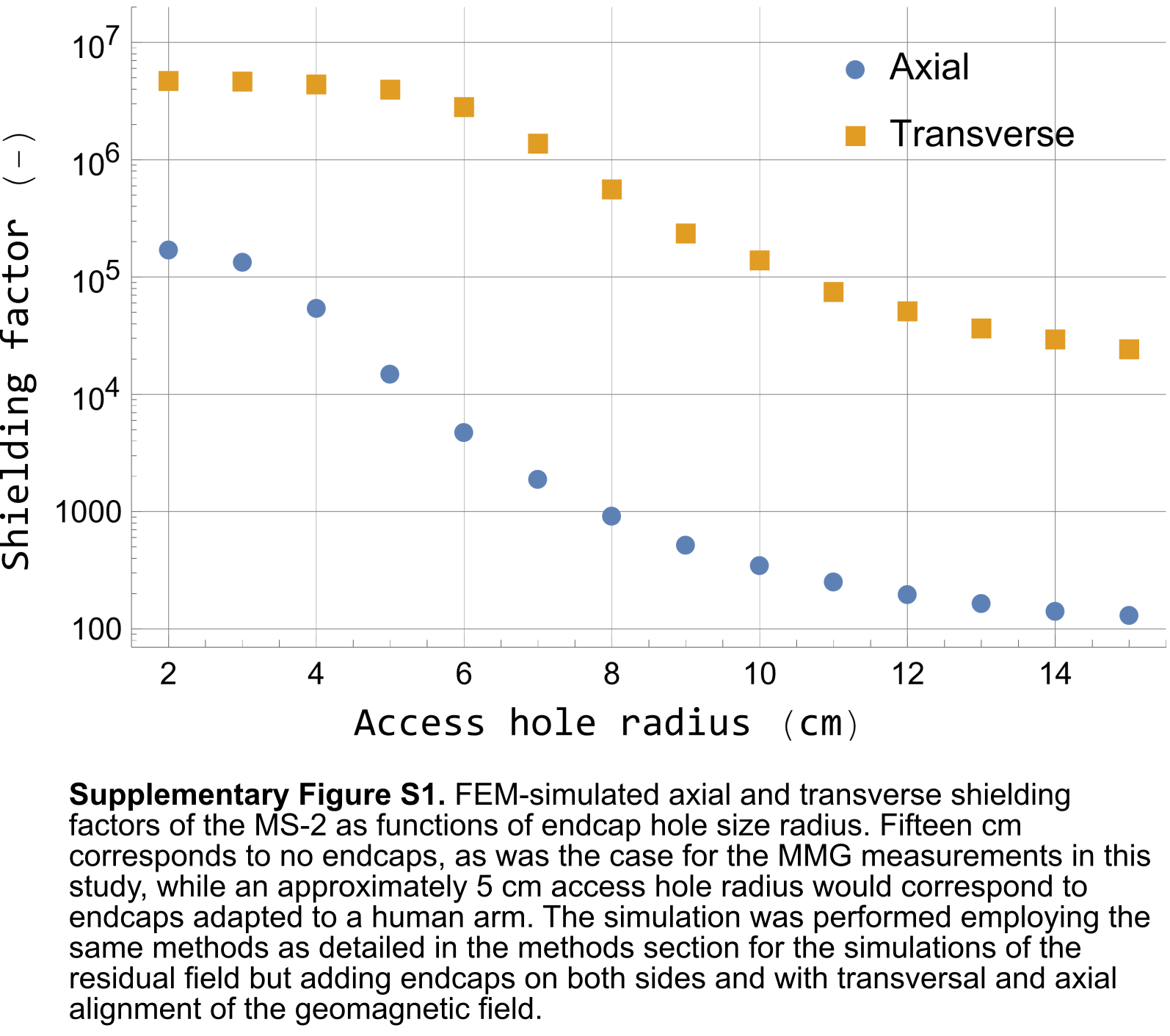

Supplement: Supplementary file 1 — Supplementary Figure S1. [file 41598_2024_69829_MOESM1_ESM.png]
